# PROCARB: A Database of Known and Modelled Carbohydrate-Binding Protein Structures with Sequence-Based Prediction Tools

**DOI:** 10.1155/2010/436036

**Published:** 2010-06-29

**Authors:** Adeel Malik, Ahmad Firoz, Vivekanand Jha, Shandar Ahmad

**Affiliations:** ^1^Biomedical Informatics Center, PGIMER, Chandigarh 160012, India; ^2^Department of Biosciences, Jamia Millia Islamia, New Delhi 110025, India; ^3^Department of Nephrology, PGIMER, Chandigarh 160012, India; ^4^National Institute of Biomedical Innovation, Department of Biomedical Research, Saito-Asagi, Ibaraki, Osaka 5670085, Japan

## Abstract

Understanding of the three-dimensional structures of proteins that interact with carbohydrates covalently (glycoproteins) as well as noncovalently (protein-carbohydrate complexes) is essential to many biological processes and plays a significant role in normal and disease-associated functions. It is important to have a central repository of knowledge available about these protein-carbohydrate complexes as well as preprocessed data of predicted structures. This can be significantly enhanced by tools *de novo* which can predict carbohydrate-binding sites for proteins in the absence of structure of experimentally known binding site. PROCARB is an open-access database comprising three independently working components, namely, (i) *Core PROCARB module*, consisting of three-dimensional structures of protein-carbohydrate complexes taken from Protein Data Bank (PDB), (ii) *Homology Models module*, consisting of manually developed three-dimensional models of N-linked and O-linked glycoproteins of unknown three-dimensional structure, and (iii) *CBS-Pred prediction module*, consisting of web servers to predict carbohydrate-binding sites using single sequence or server-generated PSSM. Several precomputed structural and functional properties of complexes are also included in the database for quick analysis. In particular, information about function, secondary structure, solvent accessibility, hydrogen bonds and literature reference, and so forth, is included. In addition, each protein in the database is mapped to Uniprot, Pfam, PDB, and so forth.

## 1. Introduction

Carbohydrates play a key role in a variety of important biological recognition processes like infection, immune response, cell differentiation, and neuronal development. All of these biological phenomena may be regulated by the interaction of these carbohydrates with proteins [1–4]. One area of therapeutic significance in protein-carbohydrate interactions has relied on the role of carbohydrates as cell surface receptors enabling adherence of bacteria, parasites, and viruses by a process known as bioadhesion [5–10]. Bacteria are often competent enough to efficiently adhere to the surface membranes of the host cells via lectin binding, thus enabling subsequent colonization and progression of the disease [11]. Irregular structure and levels of certain tumor cell surface sugars may also present opportunities for therapeutic intervention [12]. On the other hand, the ubiquitous application of carbohydrates in nature potentially poses severe specificity issues. Understanding the molecular basis of carbohydrate recognition might offer the essential basis to rationally plan biologically active saccharide analogues [13].

**Figure 1:**
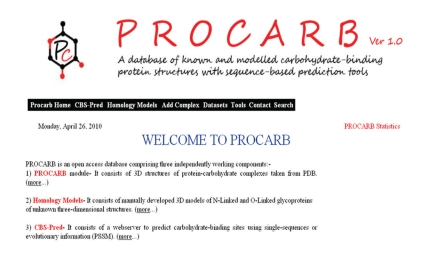
Screen shot of PROCARB Homepage. From this homepage the user can search the database with a four-letter PDB code or by a keyword search.

In spite of their numerous important biological roles, there is no appropriate database dedicated to these protein-carbohydrate complexes. Although, the Protein Data Bank (PDB) [14] stores all the experimentally determined protein-carbohydrate complexes, yet it is not easy to identify a protein-carbohydrate complex in PDB. The GLYCOSCIENCES.de web resource [15] provides numerous tools and databases which aid in searching the PDB for various carbohydrates. Moreover, the available databases like Lectines [16] & Glycoconjugate [17] databank dedicated to protein carbohydrate complexes do not have detailed information on the functionally important carbohydrate-binding residues and proteins. Hence, there is a need for a single resource where all the relevant information about a pair of interacting protein and carbohydrate would be available. Therefore, the PROCARB (Figure 1) has been developed to provide, not only a single source of annotated complexes, but also a number of precomputed features of these carbohydrate-binding proteins like solvent accessibility, secondary structure, and hydrogen bonding information. Also the role of carbohydrates in the complex is also provided in the database wherever possible. This core module consists of 604 protein-carbohydrate complexes with at least one but possibly more carbohydrate molecule(s) in each complex. Total number of carbohydrate molecules, thus is 4240, which are bound to 5360 residues in proteins.

Structure-based approach to drug design has become a standard protocol in the pharmaceutical industry where large databases of potential small drug candidates may be docked into an active site of a particular target molecule [18]. Structures of many glycoproteins of interest have not been solved yet but can be modeled because suitable templates of matching structures are available. Therefore, we have also attempted to generate the three-dimensional structures of different types of glycoproteins (both N- and O-linked), with unknown structures by using homology modelling. This module of PROCARB consists of 26 N-linked and 20 O-linked modelled structures.

Finally, functional annotation of proteins and understanding of functions in cases were only an amino acid sequence of protein is available requires predicting potential carbohydrate-binding sites, which experimentalists can then verify. Based on our previous work in this direction [19], we developed a web server which can take an amino acid sequence provided by users and predict carbohydrate-binding sites, albeit with a modest success rate keeping in view the difficulty in sequence-based prediction, which nonetheless provides useful clues for experiments.

## 2. Database Description

**Figure 2:**
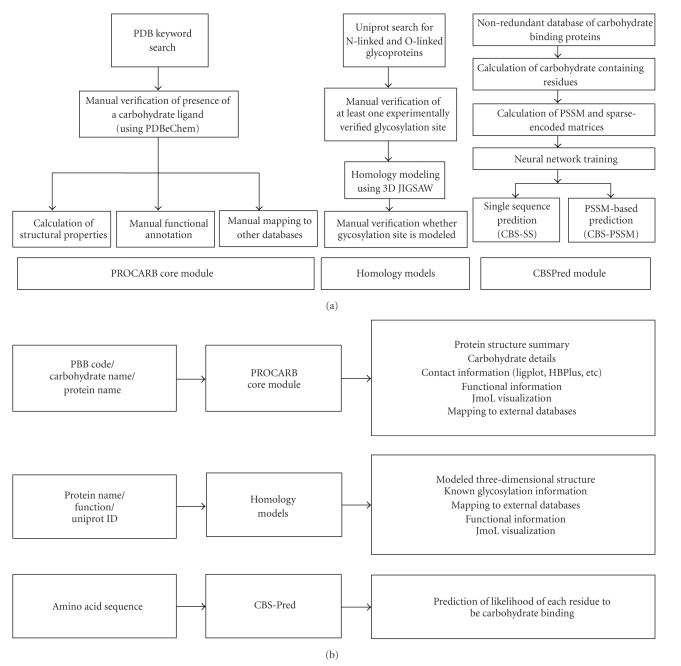
Client-side working of PROCARB modules.

Overall organization of the database is illustrated in Figures 2(a) and 2(b). As shown in the figure and stated above, the PROCARB is composed of three modules, which work largely independently. These modules are described in the following sections.

### 2.1. PROCARB Core Module

The PROCARB core module is developed by systematically locating protein-carbohydrate complexes in the protein data bank (PDB) and manual verification of existence and identification of carbohydrate ligand. A protein is considered as a carbohydrate binding if any atom of its amino acid is within a 3.5 Å cutoff distance from any atom of the sugar in the protein-carbohydrate complex [19]. Various structural and contact properties like secondary structure, hydrogen bond, van der Waal contacts, solvent accessibility, and so forth, are computed for all entries and stored in this core module of the database. In addition, a Jmol [20] visualisation is provided with preloaded scripts allowing identifying the location and nature of carbohydrate binding sites. All structures found by keyword search were validated manually for the presence of carbohydrate ligands. Specifically at the time of last update, 914 hits were obtained using keyword search in the PDB, of which only 604 proteins were found to have a carbohydrate attached, making it important that these ligands be manually annotated. The databases, so compiled, are also available for free download, both in the raw PDB file as well as a subset of entries which consists of representative structures selected at 25% sequence similarity. For each complex, the carbohydrate details were retrieved from the PDBsum [21] and to confirm whether one of the bound ligands is a carbohydrate, all ligands were manually checked either in the PDBeChem [22] database which classifies sugars as a saccharide or from the literature reference.

FASTA formatted sequences and 3D coordinates for both raw and nonredundant datasets are also stored in the database. These data sets are scheduled to be regularly updated as new entries become available from the PDB. For a quick analysis a set of four residue-wise structural features, namely, contact with carbohydrate, secondary structure, and solvent accessibility is included. These features are computed using standard software such as DSSP [23], ASAView [24], and HBPlus [25], respectively.

Information on each complex is stored in an MYSQL database where the central protein table contains information regarding the protein, its bound ligand, function, and literature reference. Web interface uses PHP and JavaScript and allows searches by a variety of text-based options like PDB code, ligand name, protein name, and source organism. Data entries are displayed using dynamically generated pages which describe the relevant information including protein name, source, ligands, Pfam [26] description Uniprot [27] ID, and so forth. Information about gene name, SCOP [28] classification, function of the protein, mutation (if any), and its attached ligands or metal ions is also provided. Information about all these proteins was extracted from various biological databases like PDB [14], Swissprot [29], and Pfam [26], and each of these entries is also directly hyperlinked to their respective entry in these databases. Precomputed structure information such as secondary structure, solvent accessibility, hydrogen bonds, and residue-carbohydrate contacts at 3.5 Å distance cutoff (using an in-house perl program) is also provided for further analysis. To help us keep the database up to date, users are encouraged to add protein-sugar complexes in the database through an online submission system. User submissions will be reviewed and added to the database after manual inspection and calculation of related properties.

### 2.2. Homology Models Module

In this module, we have attempted to generate the three-dimensional structures of a large number of glycoproteins (both N- and O-linked) with hitherto unknown structure, using automated web-based homology modeling. As a case study, a detailed project model-based 3D-structure of Hev b 4, a latex allergen N-glycoprotein has also been completed which is described elsewhere in our earlier work [30].

To select proteins for modeling, Swissprot [29] search was performed for N-linked glycoproteins using the keyword “N-linked”. O-linked glycoprotein sequences were collected from O-glycbase [31] database. To have at least one model for each protein family, the sequence data was grouped into families at 30% sequence identity and one member from each family was selected for modeling. In all cases, at least one glycosylation site was identified and annotated in Swissprot [29]. This data set has two groups each one corresponding to O-linked and N-linked glycoproteins.

Selected glycoprotein sequences, having at least one experimentally verified glycosylation site, were used as an input for the web server 3D-JIGSAW [32]. This server builds three-dimensional models for proteins on homologues of known 3D structure. The automated mode of 3D-JIGSAW [32] web server resulted in 50 homology-based models of N-linked glycoproteins out of 73 N-glycoprotein sequences and 104 structure models of O-linked glycoproteins out of initial 173 O-glycoprotein sequences. After careful examination of each model, it was noted that there were only 26 N-linked and 20 O-linked models in which at least one experimentally verified glycosylation site was modeled. Optimization of these models was carried out via CHARMm all atom forcefield minimization. Energy was minimized for a gradient of 1.0 kcal/mol by using conjugate gradient protocol available in Discovery studio version 2.0 [Accelry's Software Inc] [33] to remove any steric clashes and stabilize the models. The various types of initial potential energy, potential energy, Van der Waals energy, and electrostatic energy of N- and O-glycoprotein models after energy minimization are listed in Tables 1 and 2. Additionally, Ramachandran analysis was performed for subsequent optimization on all the 46 models using SAVES [34] web server (Tables 3 and 4). In other models, the 3D-JIGSAW [32] server was not able to model the experimentally determined glycosylation site due to the absence of a suitable template so they were not included in the web resource. Graphics highlighting the experimentally determined glycosylation sites were generated for the modeled structures using VMD [35] and form the part of database and can also be visualized in Jmol [20].

Though this is based on using automated web-based homology modeling, most of the models are within the acceptable ranges of Ramachandran score (Tables 3 and 4) and may provide some initial encouragement to use the homology models in understanding their structure-function relation by designing mutagenesis and drug designing experiments. Protein structure models can be of enormous help in functional genomics. One of the most important assistance of homology models lies in the functional genomics where they could provide structural insights to understand the protein function [36]. The 3D models have already been employed to identify the enzymatic activities [37] and ligand-binding [38] functions of proteins. Additionally, it is well known that homology modeling requires high quality of sequence alignment between the target and the template proteins; therefore, human intervention may be a possible solution for models with low scores. In spite of various limitations, homology modelling will remain an essential tool in predicting the 3D structures of proteins as the number of protein sequences will keep on increasing and it is impracticable to resolve the 3D structure of each sequence [39].

### 2.3. CBS-PRED Module

Many proteins which interact with carbohydrates (either covalently or noncovalently) are known without the knowledge of residues that participate in these interactions. Only few computational methods have been described till date which predict the covalently attached Glycosylation sites [40, 41] in proteins. Similarly, only three methods are reported for the prediction of carbohydrate binding sites in proteins based on the 3D structure of the complex [42–44]. In view of this, we have earlier developed an algorithm to identify carbohydrate-binding residues from single sequences or their evolutionary profiles [19]. CBS-Pred is an implementation of these algorithms into PROCARB. This module is made up of two submodules, namely, CBS-SS and CBS-PSSM, which utilize single sequence or alignment profiles in the backend to make a residue-wise prediction. Although PSSM-based predictions are more accurate, single sequence module is provided as a high-speed alternative as generating PSSM is time consuming. Exact performance score of these submodules is likely to change as we update neural network parameters, used for prediction with every update in training data sets. Therefore, prediction performance scores are returned with the server output and can be used to estimate the degree of false predictions.

**Figure 3:**
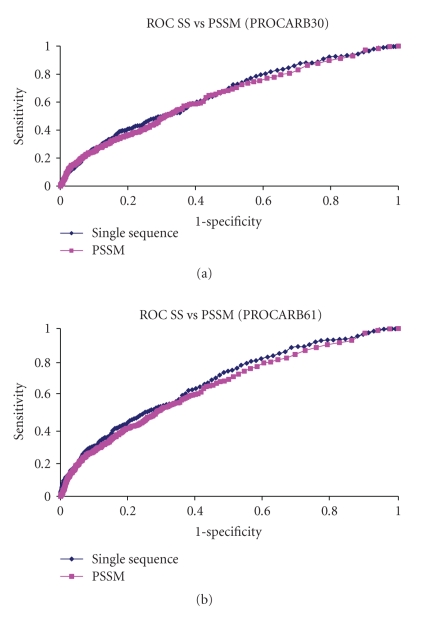
(a) Roc plot for 30 nonredundant protein carbohydrate complexes submitted to PDB between January 2007 and November 2008. (b) Roc plot for 61 protein carbohydrate complexes submitted to PDB between January 2007 and November 2008.

We also tested the CBS-Pred on Area under the ROC curve (AUC) (Table 5) for protein-carbohydrate complexes that were submitted to the PDB between January 2007 and November 2008. In this way we obtained ROC plots (Figures 3(a) and 3(b)) for the following two datasets:

- PROCARB30: A nonredundant dataset of protein-carbohydrate complexes submitted to PDB between January 2007 and November 2008.
- PROCARB61: A redundant dataset of protein-carbohydrate complexes submitted to PDB between January 2007 and November 2008.

## 3. Additional Tools

### 3.1. PROCARB BLAST

A BLAST [45] sequence similarity search has been provided which accepts user input and can search the user submitted query against the above mentioned databases. This may be helpful in determining the homologous sequences from the PROCARB database on the basis of sequence similarity.

### 3.2. Carbohydrate Finder

Due to the enormous diversity of carbohydrates, it is always difficult to identify whether a given ligand in a PDB coordinate file is a carbohydrate or not. Carbohydrate Finder identifies diverse types of carbohydrates in a given protein-carbohydrate complex. Currently, it can recognize 100 different types of carbohydrates.

### 3.3. Contact Calculator

Contact Calculator calculates the contacting pairs in a given protein-carbohydrate complex at different cutoff distances and can also recognize 100 different types of carbohydrates that may be in contact with the amino acid residues (Table 6). 

## 4. Conclusions

A database of protein-carbohydrate complexes and models of unknown glycoprotein structures was developed, and an associated sequence-based prediction module was compiled. We expect that PROCARB will facilitate functional annotation, designing of site-directed mutagenesis experiments, and modeling protein-carbohydrate interactions which in turn will help the experimental and bioinformatics research on understanding protein-carbohydrate interactions.

##  Availability and Requirements

PROCARB can be accessed at http://www.procarb.org or http://procarb.netasa.org.

## Figures and Tables

**Table 1:** Various types of energies for each N-linked model after energy minimization.

S. No.	Uniprot Id (Model)	Initial Potential Energy (kcal/mol)	Potential Energy (kcal/mol)	Van der Waals Energy (kcal/mol)	Electrostatic Energy (kcal/mol)
(1)	P02765	7202.80	−5269.26	−526.46	−5619.75
(2)	P03952	1432684.90	−13432.61	−1664.91	−13617.06
(3)	P08195	128794.29	−27001.31	−3014.99	−29181.87
(4)	P08861	37688.55	−12399.68	−1643.87	−12720.00
(5)	P08962	7860.19	−4611.32	−358.31	−5632.08
(6)	P10253	4277.54	−25010.03	−1708.90	−32940.69
(7)	P14625	334135.61	−28901.57	−3115.71	−30375.74
(8)	P15586	33799.59	−25047.64	−2548.84	−28518.54
(9)	P17346	2615.44	−4216.14	227.87	−8462.63
(10)	P19823	2306.05	−5570.83	−396.18	−7580.99
(11)	P29622	761535.82	−17395.98	−2115.57	−17857.89
(12)	P30805	809.78	−6347.63	−627.03	−7150.57
(13)	P35613	11089.12	−8539.86	−814.15	−9260.29
(14)	P49256	20519.42	−11541.44	−735.38	−14684.69
(15)	P50897	10651.77	−16006.13	−2022.72	−16075.47
(16)	P51688	302177.17	−19114.24	−1683.13	−24207.85
(17)	P52193	569701.07	−16750.64	−1694.27	−18734.06
(18)	Q13510	27411.06	−12442.43	−1252.58	−14055.71
(19)	Q13586	517.28	−2456.82	−306.03	−2525.30
(20)	Q14126	627306.18	−11241.12	−1366.40	−11832.11
(21)	Q95114	7486375.26	−17552.50	−1706.38	−19594.20
(22)	Q96PD5	9317.93	−7957.20	−1001.23	−8462.80
(23)	Q9HB40	31337.61	−21434.10	−1582.26	−25924.32
(24)	Q9HDC9	8706.65	−16186.38	−1371.38	−18996.95
(25)	Q9Y4L1	151149.78	−22399.76	−2519.80	−23513.20
(26)	Q9YGP1	6321.75	−8051.55	−921.83	−8160.34

**Table 2:** Various types of energies for each O-linked model after energy minimization.

S. No.	Uniprot Id (Model)	Initial Potential Energy (kcal/mol)	Potential Energy (kcal/mol)	Van der Waals Energy (kcal/mol)	Electrostatic Energy (kcal/mol)
(1)	P00304	1011.16	−3277.8	−107.88	−5373.8
(2)	P01217	−1564.4	−3973.3	−492.97	−4077.3
(3)	P01588	937.248	−8253.1	−991.77	−8539.6
(4)	P01866	4.1*E* + 10	−15390	−2062.7	−16107
(5)	P05451	−1936.7	−7479.7	−966.71	−7567.7
(6)	P06027	3794.54	−7010.8	−823.42	−7490.5
(7)	P06870	27038.2	−13000	−1715	−13041
(8)	P08514	140740.16	−49738.11	−4626.67	−57424.53
(9)	P26631	4.1*E* + 10	−2829.4	−263.85	−3032.5
(10)	P28314	−1098	−17977	−2188.1	−18289
(11)	P28512	165706808.06	−3414.87	−282.99	−3687.12
(12)	P36912	7304.93	−11640.89	−1207.84	−12525.97
(13)	P40225	−2638.4	−6971.1	−954.33	−7128.9
(14)	P48304	−2795	−7659	−930.64	−7811.2
(15)	P51671	76.0533	−2922.7	−436.77	−3114
(16)	P80370	−230.89	−1203.16	−102.38	−1272.45
(17)	P81054	127345.09	−19664.21	−1900.14	−21238.18
(18)	P81428	127345	−17179	−1873	−18763
(19)	P98119	3162.77	−18009.99	−1034.49	−23939.79
(20)	Q09163	15646.54	−5165.18	−500.29	−5614.36

**Table 3:** Ramachandran analysis of all N-linked models.

S. No.	Uniprot Id (Model)	Most favoured (%)	Additionally allowed (%)	Generously allowed (%)	Disallowed (%)
(1)	P02765	56.2	31.5	9.0	3.4
(2)	P03952	75.7	22.8	1.0	0.5
(3)	P08195	63.0	29.6	5.1	2.2
(4)	P08861	75.9	22.1	2.0	0.5
(5)	P08962	70.1	25.3	3.4	1.1
(6)	P10253	60.8	30.4	6.8	2.0
(7)	P14625	68.2	25.5	5.1	1.3
(8)	P15586	61.3	29.7	6.7	2.3
(9)	P17346	41.1	47.3	8.5	3.1
(10)	P19823	70.0	26.9	3.1	0.0
(11)	P29622	71.7	24.9	1.7	1.7
(12)	P30805	60.6	32.1	4.6	2.8
(13)	P35613	44.8	38.5	13.3	3.5
(14)	P49256	59.6	34.3	5.6	0.5
(15)	P50897	77.9	21.2	0.8	0.4
(16)	P51688	66.8	25.1	4.3	3.8
(17)	P52193	51.1	36.6	36.6	9.1
(18)	Q13510	64.1	29.6	4.0	2.2
(19)	Q13586	80.4	13.0	4.3	2.2
(20)	Q14126	69.9	27.5	2.6	0.0
(21)	Q95114	61.5	33.0	3.3	2.2
(22)	Q96PD5	68.9	27.4	3.0	0.7
(23)	Q9HB40	61.4	32.5	4.1	1.9
(24)	Q9HDC9	46.7	39.7	9.7	3.9
(25)	Q9Y4L1	66.9	24.4	5.9	2.8
(26)	Q9YGP1	69.4	28.2	2.4	0.0

**Table 4:** Ramachandran analysis of all O-linked models.

S. No.	Uniprot Id (Model)	Most favoured (%)	Additionally allowed (%)	Generously allowed (%)	Disallowed (%)
(1)	P00304	37.3	54.2	4.8	3.6
(2)	P01217	70.1	23.4	3.9	2.6
(3)	P01588	65.3	28.6	4.8	1.4
(4)	P01866	70.0	25.4	2.5	2.1
(5)	P05451	75.4	23.0	1.6	0.0
(6)	P06027	64.8	25.6	5.6	4.0
(7)	P06870	77.9	19.6	1.0	1.5
(8)	P08514	50.6	38.7	7.4	3.4
(9)	P26631	58.3	35.4	4.2	2.1
(10)	P28314	76.0	23.3	0.7	0.0
(11)	P28512	54.0	38.0	4.0	4.0
(12)	P36912	55.7	39.7	4.0	0.6
(13)	P40225	80.2	16.5	3.3	0.0
(14)	P48304	74.2	23.4	2.3	0.0
(15)	P51671	68.8	25.0	3.1	3.1
(16)	P80370	45.0	55.0	0.0	0.0
(17)	P81054	78.0	22.0	0.0	0.0
(18)	P81428	49.3	38.2	7.4	5.1
(19)	P98119	45.5	38.0	11.1	5.4
(20)	Q09163	46.5	36.0	8.1	9.3

**Table 5:** AUC scores for protein-carbohydrate complexes submitted to PDB between January 2007 and November 2008. Here PROCARB30-SS: single-sequence-based scores for 30 nonredundant protein-carbohydrate complexes, PROCARB30-PSSM: PSSM-based scores for 30 nonredundant protein-carbohydrate complexes, PROCARB61-SS: single-sequence-based scores for all 61 protein-carbohydrate complexes, and PROCARB61-PSSM: PSSM-based scores for all 61 protein-carbohydrate complexes.

S. no.	Dataset	AUC Scores
(1)	PROCARB30-SS	0.6518
(2)	PROCARB30-PSSM	0.637
(3)	PROCARB61-SS	0.6834
(4)	PROCARB61-PSSM	0.656

**Table 6:** Sample output from Contact Calculator exhibiting residue-carbohydrate details at atomic level.

Your uploaded file: IFV2.pdb:							
TITLE: THE HC FRAGMENT OF TETANUS TOXIN COMPLEXED WITH AN ANAL 2 OF ITS GANGLIOSIDE RECEPTOR GT1B							
The protein-carbohydrate contact details at ≤4 Å: (For all chains)							

Distance (Å)	Residue	Position	Atom	Protein-Chain	Carbohydrate		Atom Chain
3.739	ASN	219	CB	A	GAL	O6	Z
3.753	ASN	219	CG	A	GAL	O6	Z
3.937	ASN	219	OD1	A	GAL	O6	Z
3.993	ASN	219	ND2	A	NGA	C4	Z
2.813	ASN	219	ND2	A	NGA	O4	Z
3.660	ASN	219	ND2	A	NGA	O5	Z
3.922	LEU	221	CB	A	NGA	C6	Z
3.446	LEU	221	CB	A	NGA	O6	Z
3.546	LEU	221	CDl	A	NGA	C6	Z
3.827	LEU	221	CDl	A	NGA	O6	Z
***	***	***	***	***	***	***	***
***	***	***	***	***	***	***	***
3.826	TRP	289	CDI	A	NGA	CS	Z
3.826	TRP	289	CDI	A	NGA	O6	Z
3.934	TRP	289	CH2	A	SIA	O1A	Z
3.907	TYR	290	OH	A	GAL	C6	Z

The summary of Protein-Carbohydrate contact positions of your uploaded pdb file:							
Total number of contacts = 7							
At Position: 219 221 222 270 271 289 290*****							
